# The Role of the Negative Regulation of Microglia-Mediated Neuroinflammation in Improving Emotional Behavior After Epileptic Seizures

**DOI:** 10.3389/fneur.2022.823908

**Published:** 2022-04-14

**Authors:** Qiong Wu, Hua Wang, Xueyan Liu, Yajuan Zhao, Junmei Zhang

**Affiliations:** Department of Pediatrics, Shengjing Hospital of China Medical University, Shenyang, China

**Keywords:** epilepsy, microglial cells, brain injury, phagocytosis, emotional disorder, neurons

## Abstract

**Objective:**

Studies have long shown that uncontrolled inflammatory responses in the brain play a key role in epilepsy pathogenesis. Microglias play an important role in epileptic-induced neuroinflammation, but their role after epileptic seizures is still poorly understood. Alleviating epilepsy and its comorbidities has become a key area of interest for pediatricians.

**Methods:**

A pilocarpine-induced rat model of epilepsy was established. The rats were randomly divided into four groups: a control group, epilepsy group, TLR4 inhibitor group (epilepsy+TAK-242), and NF-κB antagonist group (epilepsy+BAY11–7082).

**Results:**

1. The results of TUNEL staining showed that the expression in rats in the epilepsy group was the most obvious and was significantly different from that in rats in the control, EP+BAY and EP+TAK groups. 2. The expression of TLR4 and NF-κB was highest in rats in the epilepsy group and was significantly different from that in rats in the control, EP+BAY and EP+TAK groups. 3. The fluorescence intensity and number of IBA-1-positive cells in rats in the epilepsy group were highest and significantly different from those in rats in the control, EP+BAY and EP+TAK groups. Western blot analysis of IBA-1 showed that the expression in rats in the epilepsy group was the highest and was statistically significant. 4. CD68 was the highest in rats in the epilepsy group and was statistically significant. 5. In the open-field experiment, the central region residence time of rats in the EP group was delayed, the central region movement distance traveled was prolonged, the total distance traveled was prolonged, and the average speed was increased. Compared with rats in the EP group, rats in the EP+BAY and EP+ TAK groups exhibited improvements to different degrees.

**Conclusion:**

At the tissue level, downregulation of the TLR4/NF-κB inflammatory pathway in epilepsy could inhibit microglial activation and the expression of the inflammatory factor CD68, could inhibit hyperphagocytosis, and inhibit the occurrence and exacerbation of epilepsy, thus improving cognitive and emotional disorders after epileptic seizures.

## Introduction

Epilepsy is a chronic brain disease characterized by a persistent tendency for seizures (1, 2). Epilepsy affects more than 70 million people worldwide (3), 60% of which experience childhood onset (4). In children with epilepsy, 10–30% have temporal lobe epilepsy (TLE) involving the hippocampus, amygdala, entorhinal cortex and other limbic system structures, some of which easily develop refractory epilepsy or persistent epilepsy, resulting in varying degrees of learning and memory impairment (5), cognitive dysfunction (6), and mood disorders (7, 8). However, reducing brain injury and emotional and behavioral disorders that develop after the onset of epilepsy has become a problem requiring close attention and considerable effort from pediatricians to find a solution (9).

Studies have long shown that uncontrolled inflammatory responses in the brain play a key role in the development of epilepsy (10–12). During the occurrence of TLE, temporal lobe injury is caused by the activation of microglias and astrocytes and the accumulation of inflammatory cytokines, suggesting that there is an inflammatory response in the brain during epileptic seizures (13–15). In the brains of children with intractable epilepsy (16), a large number of neurons are damaged, and microglias and astrocytes are significantly activated (17). Reducing the inflammatory response during epileptic seizures, inhibiting the activation of glial cells, and improving postseizure brain injury have become practical problems that pediatricians urgently need to solve. Thus, anti-inflammatory therapy may become a new strategy to reduce neuronal injury and reduce epileptic seizures in children (18, 19).

Microglias play an important role in neuroinflammation induced by epilepsy (20–23). They not only produce a large number of cytokines and chemokines but also seem to be the target of cytokine and chemokine signals in the central nervous system (CNS) (24–26). Inflammatory cytokines increase the excitability of neurons and are thought to contribute to the occurrence of epilepsy (27, 28). Microglias secrete specific pro-inflammatory factors in the brain, and the increased expression of the M1 phase cytokine CD68 reflects the strong phagocytic ability of microglias. Toll-like receptor (TLR) signaling is also involved in cytokine production in epileptic models (29). Studies have shown that the activated TLR4 pathway (mediated by MyD88) is part of the molecular response that promotes a pro-inflammatory environment after status epilepticus (SE) (30), but the specific mechanism of activation of inflammatory factors needs further verification.

Microglias mediate macrophages in brain nerve inflammation. When microglias detect injury, they quickly migrate to damaged areas and trigger the activation of cascades (31); importantly, microglias may cause neuronal injury. Astrocytes are involved in this process, and the dysfunction of astrocytes in the brains of epileptic patients has been well-studied, but the role of excessive phagocytosis of microglias in inducing epilepsy and causing brain damage in epilepsy is still largely uncertain (32).

## Materials and Methods

### Animals

A total of 88 rats healthy male Sprague-Dawley rats (from the Medical Animal Center, Shengjing Hospital of China Medical University), 3 weeks of age and weighing 45.0–60 g, were used in this study.After adapting to the experimental environment, the rats were fed in a standard 12/12 h alternating light and dark animal room at 22–24°C, with a relative humidity of 50 ± 10%, and were provided with ample nutritious food and free access to drinking water.

### Experimental Methods

#### Establishment of the Rat Model of SE and the Experimental Groups

##### Modeling Method

The model in the present study was based on the rat model of SE established by Rossi et al. Healthy male SD rats in the experimental group were first intraperitoneally injected with 3 mEg/kg lithium chloride (127 mg/kg) and then intraperitoneally injected with 1 mg/kg atropine sulfate 18–20 h later. Thirty minutes after an atropine injection, 30 mg/kg pilocarpine was intraperitoneally injected, and seizures were closely observed. After 30 min, 10 mg/kg pilocarpine was intraperitoneally injected. This protocol was repeated every 30 min until the onset of SE. The control group was injected with the same volume of saline. More than half an hour after SE was established, 10 mg/kg diazepam was intraperitoneally injected, and SE was controlled to reduce rat death. Spontaneous epileptic seizures repeatedly occurred in the rats in the chronic stage, and the seizures spontaneously stopped after more than 1 min. No antiepileptic drugs were administered to control the seizures. The Racine (33) grading standard was used to evaluate epileptic seizures in experimental animals. According to the Racine behavioral scoring table, status epilepticus was defined as a grade IV-V convulsive state and convulsive state for at least 30 min, and thus these rats were included in the model group with status epilepticus. Rats that did not present grade IV seizures were excluded from the model group.

The rats (*n* = 88 total) were divided into four groups (*n* = 22 per group): I. Rats in the normal control group were injected with a volume of saline matching the volume of substances injected into rats in experimental groups at the corresponding time point. II. Pilocapine was used to establish SE in SD rats. Samples were collected 2 days after the corresponding model was established. III. SD rats were treated by intraperitoneal injection of a nuclear factor kappa B (NF-κB) inhibitor (BAY 11–7082) in solvent. Rats in the NF-κB inhibitor group were intraperitoneally injected once a day with 5 mg/kg BAY11–7082 (Sigma, USA) 1 h after the model was established and for the next 2 days (BAY11–7082 was dissolved in dimethyl sulfoxide (DMSO) solution and then diluted in corn oil, and the final concentration of DMSO solution was 1%); IV. The solvent-treated SD rats were intraperitoneally injected with a TLR4 inhibitor (TAK-242). The selective inhibitor of TLR4, TAK-242 (Princeton, USA), was intraperitoneally injected into rats at a dose of 3 mg/kg 1 h after the model was established and once a day for the following 2 days (TAK-242 was dissolved in DMSO solution and then diluted in corn oil until the concentration of DMSO solution was 1%). Samples were collected from rats in these four groups 2 days after successful establishment of the model and treatment. Brain tissue samples were collected from six rats for Nissl staining and double immunofluorescence staining. Hippocampal tissues were extracted from the other six rats for Western blotting and polymerase chain reaction (PCR) analysis (*n* = 6 per group). The cognitive function test and open-field test (OFP) were carried out with a Morris water maze 7 days after successful establishment of the model (*n* = 10 per group). This maze is used to measure general motor ability, intelligence, and anxiety-related emotional behavior.

#### TUNEL Staining

Sections were dewaxed until water permeated them. Fifty microliters of 0.1% Triton X−100 (0.1% sodium citrate) was added to the sections, and they were incubated at room temperature for 8 min. Next, 50 μl of the terminal deoxynucleotidyl transferase dUTP nick end labeling (TUNEL) reaction solution was added dropwise and incubated in a humid chamber in the dark at 37°C for 60 min. Sections were subsequently stained with 4',6-diamidino-2-phenylindole (DAPI) for 5 min. A fluorescence quenching agent was added to the slices, followed by a coverslip. Microscopically, staining was observed under a fluorescence microscope, and photos were taken.

#### Immunofluorescence Staining

The sections were dewaxed in water, and citric acid repair solution was used for antigen retrieval. Non-specific antigen binding was blocked by incubation with goat serum for 40 min, and then sections were incubated overnight in a refrigerator at 4°C in the following primary antibodies: 100 μl of the prediluted primary antibodies against ionized calcium-binding adapter molecule 1 (Iba-1) (anti-mouse IgG diluted 1:80, Santa Cruz, USA), TLR4 (anti-rabbit IgG diluted 1:100, Novus, USA), and NF-κB (anti-rabbit IgG diluted 1:100, Proteintech, USA). The fluorescent goat anti-rat and anti-rabbit secondary antibodies were incubated with the sections (diluted 1:500) for 4 h in the dark at room temperature, the nuclei were stained with DAPI, and the slices were sealed. The images were collected using a confocal microscope. In each section, different locations of the CA1, CA3, and dentate gyrus (DG)3 in the hippocampus were randomly selected. The images were captured at 400x magnification and stored.

#### Immunohistochemistry

After dewaxing the sections, hydrating the sections, incubating the sections with the citric acid antigen repair solution, blocking endogenous peroxidase activity, and blocking nonspecific antigens, 1 to 2 drops of goat serum (reagent B) were added to each section; then, the sections were incubated at room temperature for 40 min. Fifty microliters of the CD68 antibody (anti-mouse IgG, diluted 1:100, Novus, USA) was added to the sections dropwise and incubated overnight in a refrigerator at 4°C. Sections were incubated with the secondary antibody, and then the sections were incubated with 1 drop of reagent C and 1 drop of reagent D for 20 min for the enzyme-substrate reaction. For color development, 50 μl of 3,3′-diaminobenzidine (DAB) working solution was added to the sections and incubated at room temperature for 20's. After staining with hematoxylin for 10 s, the color of the samples returned to blue, and the samples were then rinsed with water; sections were then dehydrated, rendered transparent, and sealed. Images of immunohistochemical staining were captured at 400 × magnification and stored.

#### Western Blotting

The hippocampal tissues were extracted from frozen separate single animal extracts each used for western analysis. Tissue samples were weighed, lysis solution were added, the samples were fully lysed, and the protein concentration of the samples were determined. Then, the samples were mixed well with 5X loading buffer, heated to denature the protein, and cooled to room temperature prior to use. A 12.5% gel was prepared for electrophoresis. Electrophoresis buffer, transfer buffer and blocking solution were prepared. Samples were loaded on the gel, separated by electrophoresis, transferred to a membrane, blocked, and incubated with a primary antibody [Iba-1 diluted 1:400, anti-mouse IgG, Santa Cruz, USA; GAPDH diluted 1:2000, anti-rabbit IgG, Proteintech, USA; CD68 (diluted 1:500, anti-mouse IgG, Novus, USA; TLR4 diluted 1:500, anti-rabbit IgG, Novus, USA), and NF-κB (diluted 1:500, anti-rabbit IgG, Proteintech, USA)] overnight at 4°C. The membrane was incubated with a goat anti-rabbit IgG secondary antibody (1:10000) and a goat anti-mouse secondary antibody (1:10000) to allow visualization of the antibody targets, and images were captured. The gray value of the Western blot results was analyzed using ImageJ software. The gray value of the target protein was normalized to the gray value of the internal reference protein for analysis.

#### Fluorescence Quantitative PCR

Total RNA was extracted from hippocampal tissue with TRIzol reagent followed by the removal of genomic DNA from brain mRNA, reverse transcription (RT) and PCR amplification. The following primer sequences were prepared.

TLR4 forward primer 5′-CAGAATGAGGACTGGGTGAG-3′, reverse primer 5′-GTTGGCAGCAATGGCTACAC-3′; NF-κB forward primer 5′-CCAAAGACCCACCTCACC-3′, reverse primer 5′-TGGCTAATGGCTTGCTCC-3′; and β-actin forward primer 5′-GGAGATTACTGCCCTGGCTCCTAGC-3′, and reverse primer 5′-GGCCGGACTCATCGTACTCCTGCTT-3′. The 2^−Δ*ΔCt*^ values were calculated to indicate the relative gene mRNA expression level and to analyze the results.

#### Open-Field Test

The OFP is a common experimental method to detect the emotional behavior of animals. The device consists of an open-field reaction box and an automatic data acquisition and processing system. The size of the open-field reaction box was 100 × 100 × 40 cm (length × width × height). The box was constructed from medical ABS material, the inner wall was painted black, and a digital camera was placed 2 m above it. The distance traveled by the rats in the reaction chamber, the number of times the rats crossed the central area (50 × 50 cm) and the time the rats stayed in the central area within 10 min were observed.

### Statistical Analysis

GraphPad Prism 7.0 analysis software was used for data processing and statistical analysis. Measurement data are presented as the means ± standard deviations (means±SD). A t test was used to compare data between two groups, and one-way analysis of variance was used to compare data among multiple groups. *P* < 0.05 was considered statistically significant.

## Results

### TUNEL Staining

**Figure 1:**
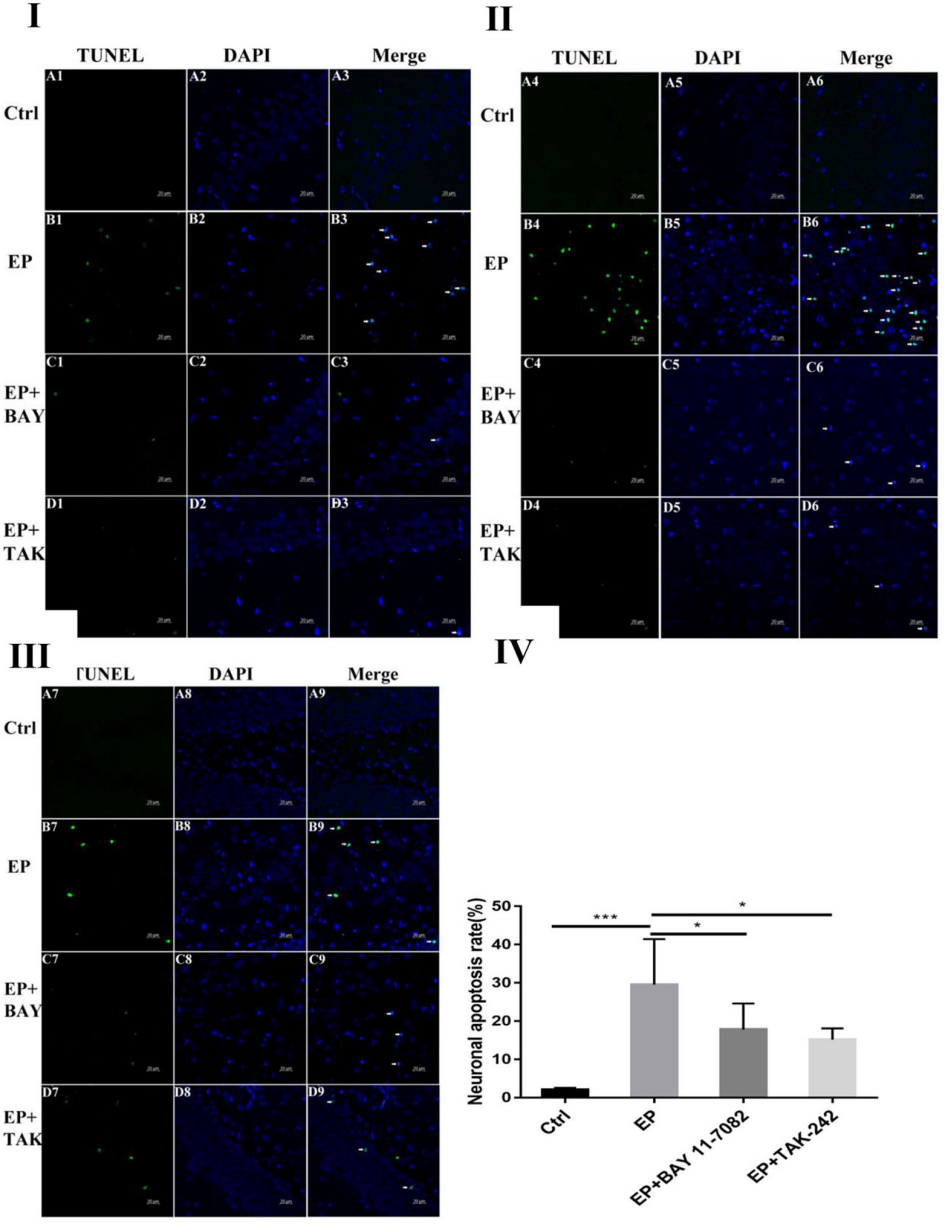
Neuronal damage observed in the hippocampal region (× 400). *P < 0.05; **P < 0.01; ***P < 0.001.

Damage to neurons in the hippocampal CA1, CA3 and DG regions was observed (Figures 1I,II,III). In rats in the control group, the hippocampal neuron bands were arranged neatly, with uniform staining and blue nuclei, and dead neurons were not observed or were only noted occasionally (Figure 1A1–9). In rats in the epilepsy (EP) group, after continuous SE, TUNEL-positive cells appeared in each area of the hippocampus in 2 days, and the nuclei were green (Figure 1B1–9). In rats in the EP+BAY group (Figure 1C1–9) and EP+TAK group (Figure 1D1–9), there were fewer TUNEL-positive cells in each area of the hippocampus at 2 d than in rats in the EP group.

Figure 1I Neuronal damage observed in the hippocampal CA1 region (400 ×, scale bar: 20 μm). Figure 1II Observation of hippocampal CA3 neuron damage. Figure 1III Neuronal damage observed in the hippocampal DG region (400 ×, scale 20 μm). Figure 1IV Statistical analysis of the percentage of hippocampal neuron death observed in each group. Figure 1 shows green TUNEL-positive cells in Figure 1A1–D1,A4–D4,A7–D7; blue DAPI nuclear staining in Figure 1A2–D2,A5–D5,A8–D8; and colocalization of TUNEL-positive cells and DAPI in Figure 1A3–D3,A6–D6,A9–D9. Figure 1A1–9 shows data from animals in the control group, and B1–B9 shows data from animals in the epilepsy group. Changes in neuronal damage in the hippocampal CA1, CA3, and DG areas in rats in the EP+BAY group (Figure 1C1–9) and EP+TAK group (Figure 1D1–9). Figure 1IV the detection results showed that the expression in rats in the epilepsy group was the most obvious (*P* < 0.001) compared with that in rats in the control group, and there was a significant difference between rats in the epilepsy group and the EP+BAY group and EP+TAK group (*P* < 0.05, *n* = 6).

### Role of the Downregulated TLR4/NF-κB Inflammatory Pathway in Epilepsy

#### TLR4 Expression in the Hippocampus of Animals in Each Group After Downregulation of the TLR4/NF-κB Inflammatory Pathway

**Figure 2:**
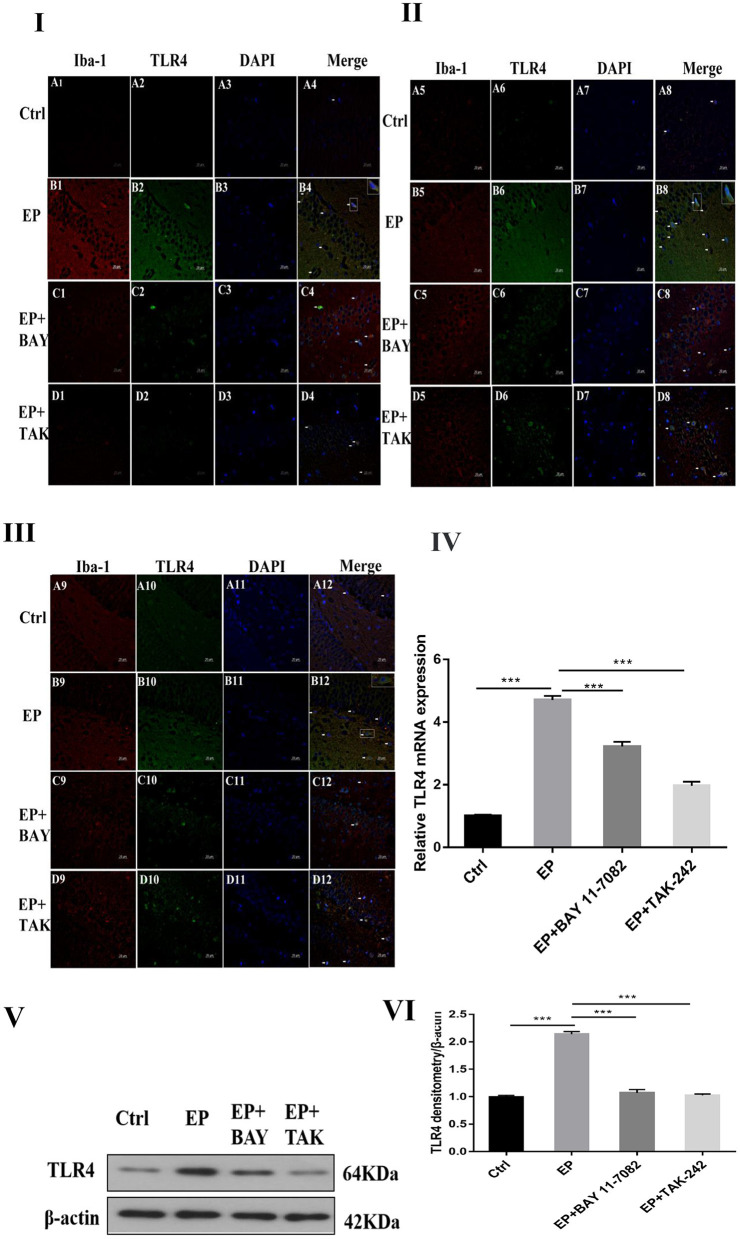
TLR4 expression in rats in each group after I downregulated the TLR4/NF-κB inflammatory pathway. *P < 0.05; **P < 0.01; ***P < 0.001.

The colocalization results of double immunofluorescence for TLR4 and the microglial marker IBA-1 were as follows: double immunofluorescence revealed colocalization of the microglial markers IBA-1 and TLR4 in the control group, epilepsy (EP) group, epilepsy+BAY 11–7082 (EP+BAY) group, and epilepsy+TAK242 (EP+ TAK) group. Two days after SE, TLR4 was expressed in microglias in the hippocampal CA1, CA3, and DG areas in rats in the control group, but the expression of TLR4 was significantly increased in rats in the EP group. The expression of TLR4 in rats in the EP+BAY and EP+TAK groups was lower than that in rats in the EP group (see Figures 2I,II,III).

Changes in TLR4 mRNA expression in hippocampal tissues were detected by RT–PCR. The results were as follows: The mRNA expression changes in TLR4 relative to the internal reference β-actin are shown in Figure 2IV After 2 days, results for rats in the control group, EP group, EP+BAY group and EP+TAK group were significantly different. The expression in rats in the EP group was the highest and was significantly different from that in rats in the control group, EP+BAY group and EP+TAK group (^*^P < 0.05; ^**^P < 0.01; ***P < 0.001). The detection results showed that the expression of rats in the EP group was the highest and was significantly different compared with that of rats in the control group, EP+BAY group and EP+TAK group (*P* < 0.001, *n* = 6).

Western blot analysis of TLR4 protein expression in the hippocampus of rats in each group showed the following.

The molecular weights of TLR4 and β-actin were 64 and 42 kD, respectively (Figure 2V). After 2 days, results for rats in the control group, EP group, EP+BAY group and EP+TAK group were significantly different. The expression in rats in the EP group was the highest and was significantly different from that in rats in the control group, EP+BAY group and EP+TAK group (^*^P < 0.05; ^**^P < 0.01; ^***^P < 0.001) (Figure 2VI). The detection results showed that the expression in rats in the EP group was the highest and was significantly different compared with that in rats in the control group, EP+BAY group and EP+TAK group (*P* < 0.001, *n* = 6).

In Figure 2I, the colocalization results of double immunofluorescence TLR4 and microglias marker IBA-1 in the hippocampal CA1 region of rats in each group are shown. Figure 2II shows the TLR4 expression in the hippocampal CA3 region of rats in each group. Figure 2III depicts TLR4 expression in the hippocampal DG of rats in each group; (400 ×, scale 20 μ m). In Figure 2IV, changes in TLR4 mRNA expression of rats in each group are shown. In Figure 2V, the expression changes in TLR4 and internal reference β-actin protein are shown. Figure 2VI shows the relative expression levels of TLR4 in rats in the control group, EP group, EP+BAY group and EP+TAK group relative to the internal reference β-actin and compares the differences. In Figure 2, red IBA-1, green TLR4 staining, blue DAPI nuclear staining are depicted. Figure 2A1–D1,A5–D5,A9–D9 shows IBA-1-positive cells; Figure 2A2–D2,A6–D6,A10–D10 depict TLR4-positive cells in green; Figure 2A3–D3,A7–D7,A11–D11 shows blue DAPI nuclear staining; Figure 2A4–D4,A8–D8,A12–D12 display colocations of positive cells for IBA-1, TLR4 and DAPI. Figure 2IA1–4 shows data for animals in the control group and B1–B4 shows data for animals in the EP group, EP+BAY group (Figure 2C1–4) and EP+TAK group (Figure 2D1–4) demonstrating changes in the expression of TLR4 in microglias in the hippocampal CA1 region. Figure 2IIA5–8 shows data for animals in the control group, B5–B8 shows data for animals in the EP group, EP+BAY group (Figure 2C5–8) and EP+TAK group (Figure 2D5–8) demonstrating changes in the expression of TLR4 in microglias in the hippocampal CA3 region. Figure 2A9–12 shows data for animals in the control group B9–B12 show data for animals in the EP group. Changes in TLR4 expression in microglias in the DG area of the hippocampus in the EP+BAY group (Figure 2C9–12) and EP+TAK group (Figure 2D9–12) are shown.

#### NF-κB Expression in the Hippocampus of Each Group After Downregulation of the TLR4/NF-κB Inflammatory Pathway

The colocalization results of dual immunofluorescence for NF-κB and the microglial marker IBA-1 were as follows.

**Figure 3:**
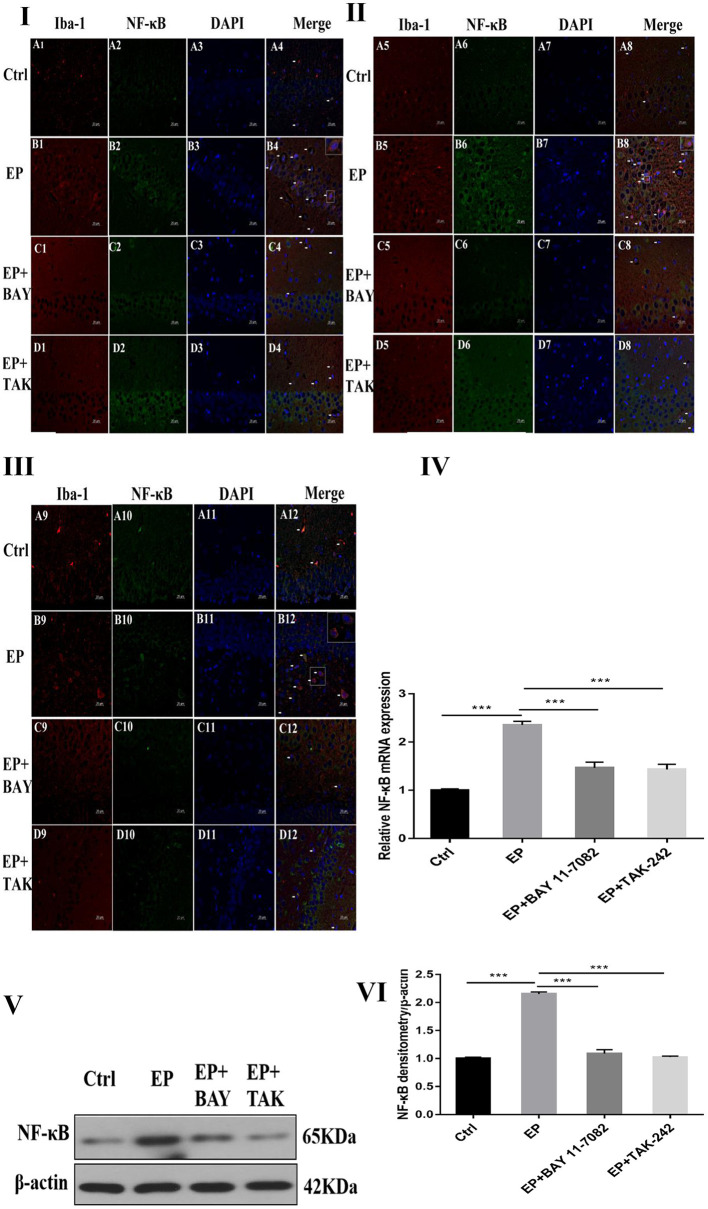
NF-κB expression after I downregulated the TLR4/NF-κB inflammatory pathway. *P < 0.05; **P < 0.01; ***P < 0.001.

Dual immunofluorescence for NF-κB and the microglial marker IBA-1 in rats in the control group, EP group, epilepsy+BAY 11–7082 (EP+BAY) group, epilepsy+TAK242 (EP+ TAK) group, 2 days after SE, NF-κB expression in hippocampal CA1, CA3, and DG microglias were low in rats in the control group, and NF-κB expression was significantly increased in rats in the EP group. NF-κB expression in rats in the EP+BAY group and EP+TAK group was lower than that in rats in the EP group (see Figures 3I,II,III).

The mRNA expression of NF-κB in the hippocampus was detected by RT–PCR. The results were as follows: The relative mRNA expression of NF-κB and internal reference β-actin is shown in Figure 3IV. After 2 days, results for rats in the control group, EP group, EP+BAY group and EP+TAK group were significantly different. The expression in rats in the EP group was the highest, which was significantly different from that of rats in the control group, EP+BAY group and EP+TAK group (^*^P < 0.05; ^**^P < 0.01; ^***^P < 0.001). The detection results showed that the expression in rats in the EP group was the highest, with a significant difference compared with that of rats in the control group, EP+BAY group and EP+TAK group (*P* < 0.001, *n* = 6).

Changes in NF-κB protein expression in the hippocampi of rats in each group were detected using Western blotting. The molecular weights of NF-κB and β-actin were 65 and 42 kD, respectively. Two days after epilepsy, there were differences among rats in the control group, EP group, EP+BAY group, and EP+TAK group (Figure 3V). The expression of rats in the EP group was the highest, and there was a significant difference compared with rats in the control group, EP+BAY group and EP+TAK group (^*^P < 0.05; ^**^P < 0.01; ^***^P < 0.001) (Figure 3VI). The detection results showed that the expression of rats in the EP group was the highest and was significantly different compared with that of rats in the control group, EP+BAY group and EP+TAK group (*P* < 0.001, *n* = 6).

Figure 3I NF-κB expression in the hippocampal CA1 region after I downregulated the TLR4/NF-κB inflammatory pathway. Figure 3II shows NF-κB expression in the hippocampal CA3 region after I downregulated the TLR4/NF-κB inflammatory pathway. Figure 3III shows NF-κB expression in the hippocampal DG region after I downregulated the TLR4/NF-κB inflammatory pathway (400 ×, scale 20 μm). Figure 3IV indicates the relative expression levels of NF-κB mRNA in rats in the control group, EP group, EP+BAY group and EP+TAK group relative to the internal reference β-actin and the comparative differences. In Figure 3V, the expression of NF-κB and the internal reference β-actin is shown. Figure 3IV shows the relative expression levels of NF-κB in rats in the control group, EP group, EP+BAY group, and EP+TAK group relative to the internal reference β-actin and compares the differences. In Figure 3, red IBA-1, green NF-κB staining, blue DAPI nuclear staining are shown. In Figure 3A1–D1,A5–D5,A9–D9 red indicates cells positive for IBA-1. Figure 3A2–D2,A6–D6,A10–D10 shows cells positive for NF-κB in green. Figure 3A3–D3,A7–D7,A11–D11 shows blue DAPI nuclear staining. Figure 3A4–D4,A8–D8,A12–D12 depict colocation of positive cells for IBA-1, NF-κB, and DAPI. Figure 3IA1–4 shows data for animals in the control group, B1–B4 in the EP group, EP+BAY group (Figure 3C1–4) and EP+TAK group (Figure 3D1–4) regarding changes in the expression of NF-κB in microglias in the hippocampal CA1 region. Figure 3IIA5–8 shows data for animals in the control group, Figure 3B5–B8 shows data for animals in the EP group, EP+BAY group (Figure 3C5–8) and EP+TAK group (Figure 3D5–8) regarding changes in the expression of NF-κB in microglias in the hippocampal CA3 region. Figure 3A9–12 shows data for animals in the control group; Figure 3B9–B12 shows data for animals in the EP group. Changes in NF-κB expression in microglias in the DG area of the hippocampus in the EP+BAY group (Figure 3C9–12) and EP+TAK group (Figure 3D9–12) are shown.

### Activation and Polarization of Hippocampal Microglias in Each Group After Downregulation of the TLR4/NF-κB Inflammatory Pathway

#### Expression of IBA-1 Protein in Hippocampal Microglias in Each Group After Downregulation of the TLR4/NF-κB Inflammatory Pathway

**Figure 4:**
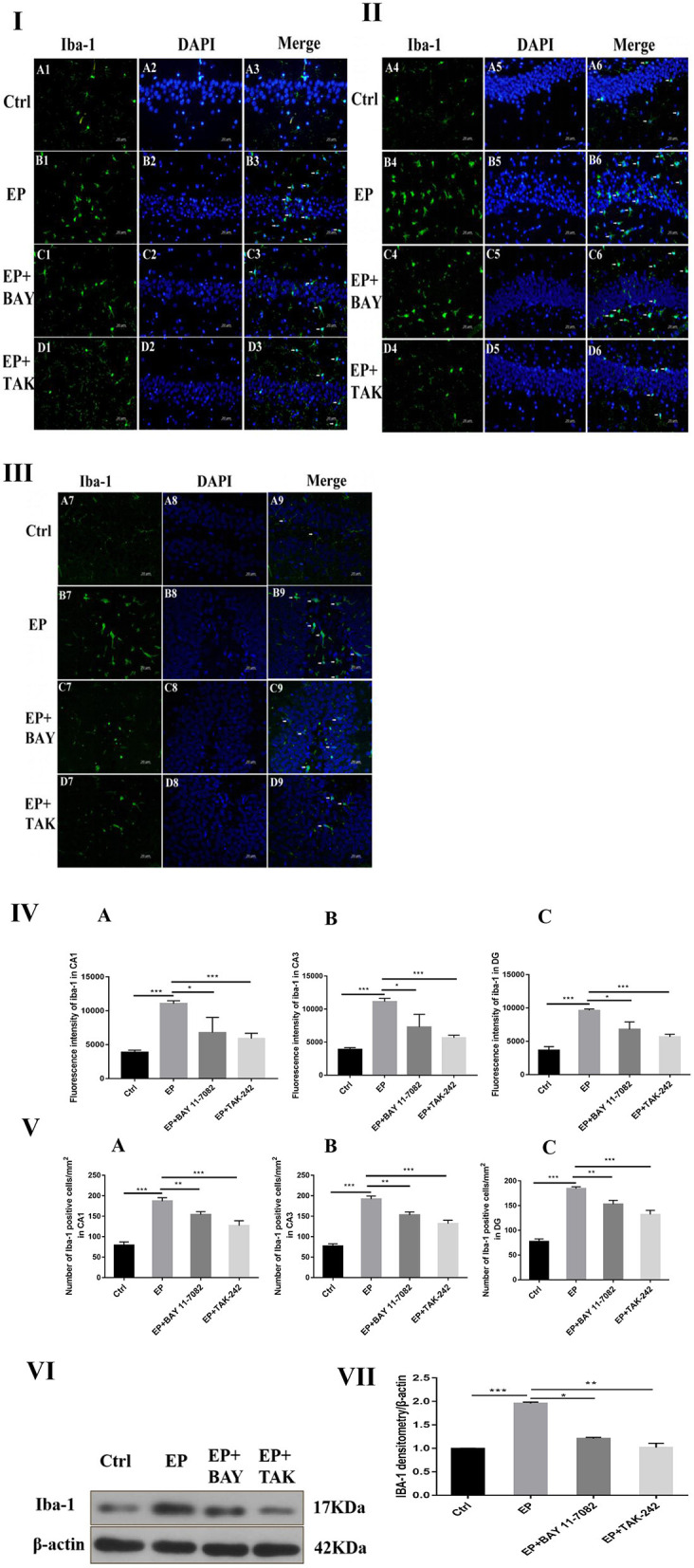
Expression of IBA-1 protein in each group. *P < 0.05; **P < 0.01; ***P < 0.001.

The expression of microglias in the hippocampus of each group was observed by fluorescence staining as follows: Fluorescence staining was used to observe the changes in the morphology and number of microglias in the hippocampal CA1, CA3, and DG regions of each group (Figures 4I,II,III). In rats in the control group, the hippocampal neuron bands were arranged neatly, with uniform staining, and the nuclei were blue. Occasionally, the microglias were seen as rod-shaped branches that were stained fluorescent green (Figure 4A1–9). In rats in the EP group, the number of microglias increased, and the cell volume increased in each area of the hippocampus 2 days after continuous epilepsy (SE), showing an amoebal shape, green fluorescence staining and blue nuclei (Figure 4B1–9). Compared with rats in the EP+BAY group (Figure 4C1–9) and EP+TAK group (Figure 4D1–9), the number of IBA-1-positive cells in each area of the hippocampus decreased at 2 days compared with that in rats in the EP group. The morphology of microglial cells decreased to different extents compared with those of rats in the EP group, and the number of activated microglias decreased. After treatment, most microglias were in a branched, rod-like, resting state.

Compared with that in rats in the control group, the fluorescence intensity of IBA-1 in rats in the EP group was increased (*P* < 0.001), the fluorescence intensity of IBA-1 in rats in the EP+BAY 11–7082 group was slightly decreased (*P* < 0.01), and the fluorescence intensity of IBA-1 in rats in the EP+TAK-242 group was decreased (*P* < 0.001). The condition of rats in the EP+BAY 11–7082 and EP+TAK-242 groups improved to varying degrees (Figure 4). The number of microglias increased (*P* < 0.001) in rats in the EP+BAY 11–7082 group compared with that in rats in the EP+BAY 11–7082 group, and the number of microglias decreased (*P* < 0.05). In rats in the EP+ TAK-242 group, the number of microglial cells in rats in the IBA-1 unit area mm^2^ decreased by *P* < 0.001, and rats in EP+BAY 11–7082 and EP+ TAK-242 groups showed different degrees of improvement, as depicted in Figure 4.

The expression of IBA-1 protein in the hippocampus was detected by Western blot.

The molecular weights of Iba-1 and β-actin were 17 and 42 kD, respectively. After 2 days, results for rats in the control group, EP group, EP+BAY group and EP+TAK group were significantly different. The expression in rats in the EP group was the highest, which was significantly different from that in rats in the control group, EP+BAY group and EP+TAK group. The detection results showed that the expression in rats in the EP group was the highest, with *P* < 0.001, compared with that of rats in the control group, P < 0.05, compared with that of rats in the EP+BAY group, *P* < 0.01, and compared with that of rats in the EP+TAK group.

Figure 4I shows protein expression of IBA-1 in microglias of the hippocampal CA1 region in rats in each group. Figure 4II shows protein expression of IBA-1 in microglias of the hippocampal CA3 region in rats in each group. Figure 4III shows protein expression of IBA-1 in microglias of the hippocampal DG region in rats in each group (400 ×, scale bar = 20 μm). Figure 4IV shows the statistical analysis of the fluorescence intensity of microglial IBA-1 in the hippocampus of rats in each group. Figure 4A shows the statistical analysis of data for each group in the CA1 region, Figure 4B shows that for the CA3 region and Figure 4C shows the statistical analysis of data for each group in the DG region. Figure 4V depicts the results of the statistical analysis of the number of IBA-1-positive microglial cells in each region of the hippocampus in rats in each group. A shows the CA1 region, B shows the CA3 region, and C shows the DG region. Figure 4VI shows the protein expression changes in IBA-1 and the internal reference β-actin. Figure 4VII shows the relative expression levels of IBA-1 in rats in the control group, EP group, EP+BAY group and EP+TAK group relative to the internal reference β-actin and compares the differences. In Figure 4A1–D1,A4–D4,A7–D7, green indicates cells positive for IBA-1. In Figure 4A2–D2,A5–D5,A8–D8, blue indicates nuclear DAPI staining. In Figure 4A3–D3,A6–D6,A9–D9, colocation of IBA-1-positive cells and DAPI is shown (*n* = 6, ^*^P < 0.05; ^**^P < 0.01; ^***^P < 0.001).

#### Expression of CD68 Protein in Hippocampal Microglias in Rats in Each Group After Downregulation of the TLR4/NF-κB Inflammatory Pathway

The expression of CD68 in the hippocampus was detected by Western blot.

The molecular weights of CD68 and β-actin were 100 and 42 kD, respectively. After 2 days, results for rats in the control group, EP group, EP+BAY group and EP+TAK group were significantly different. The expression in rats in the EP group was the highest and was significantly different from that in rats in the control group, EP+BAY group, and EP+TAK group.

**Figure 5:**
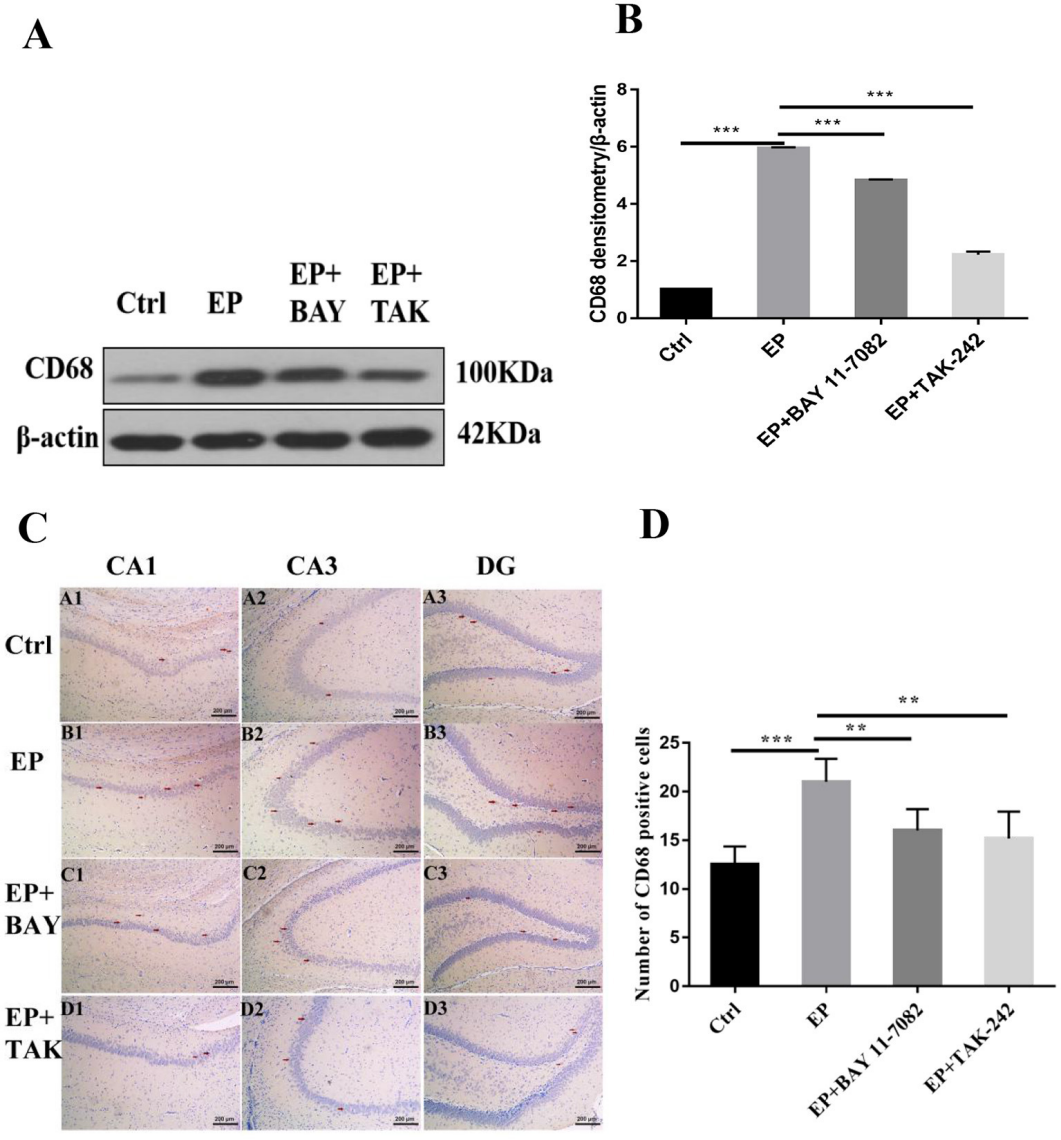
**(A–D)** Changes in CD68 protein expression in the hippocampus of rats in each group after SE seizures. *P < 0.05; **P < 0.01; ***P < 0.001.

Immunohistochemical results showed that CD68 presented granular dispersion in the hippocampal CA1, CA3, and DG regions (Figure 5C). Immunohistochemical CD68-positive cell analysis showed that the expression in rats in the EP group was higher than that in rats in the control group, EP+BAY group and EP+TAK group (Figure 5D).

Figure 5A shows the change in CD68 and internal reference β-actin protein expression. Figure 5B shows the relative expression levels of CD68 in rats in the control group, EP group, EP+BAY group, and EP+TAK group relative to the internal reference β-actin and compares the differences. Figure 5C shows CD68 immunohistochemistry. Red arrows indicate CD68-positive cells (100 ×, scale bar 200 μm). Figure 5D shows the statistical analysis results of CD68-positive cells in rats in each group. The detection results showed that the expression of rats in the EP group was the highest among the CD68 indicators, and there was a significant difference (*P* < 0.01) between rats in the EP group and the control group and between rats in the EP+BAY group and EP+TAK group (*n* = 6, ^*^P < 0.05; ^**^P < 0.01; ^***^P < 0.001).

#### The Effects of Each Group's Condition on the Emotional and Behavioral Functions of Rats Were Detected Using an Open-Field Assessment

**Figure 6:**
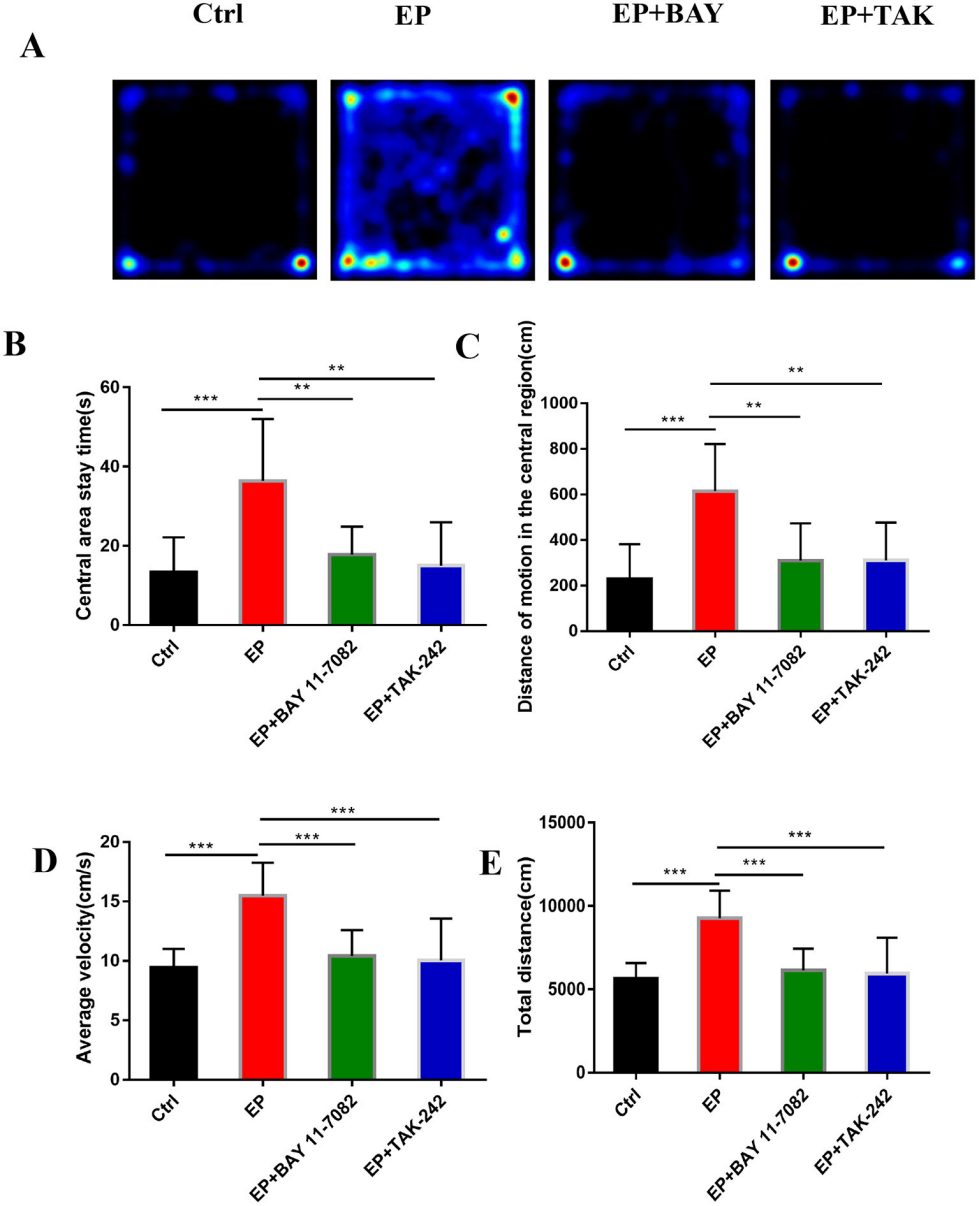
**(A–E)** The effect of each group's condition on the emotional and behavioral functions of rats detected using the OFP. *P < 0.05; **P < 0.01; ***P < 0.001.

Figure 6A shows the behavioral trajectories of rats in each group in the open-field experiment. Figure 6B shows the statistical analysis of the residence time in the central region of each group of rats in the open-field experiment. Figure 6C shows the statistical analysis of the movement distance in the central region of each group of rats in the open-field experiment. Figure 6D shows the statistical analysis of the total movement distance traveled by rats in the open-field experiment. Figure 6E shows the statistical analysis of the average velocity of rats in each group in the open-field experiment. The results showed that the central region residence time of rats in the EP group was delayed (*P* < 0.001), the central region movement distance was prolonged (*P* < 0.001), the total distance was prolonged (*P* < 0.001), the average speed was increased (*P* < 0.001), and the central region residence time of rats in the EP+BAY 11–7082 group was shortened (*P* < 0.01) compared with the outcomes for rats in the EP group. Compared with the outcomes for rats in the EP+ TAK-242 group, the median residence time of rats in the EP+TAK-242 group was shorter (*P* < 0.01), the median distance traveled by rats in the EP+ TAK-242 group was shorter (*P* < 0.01), the median distance traveled by rats in the EP+ TAK-242 group was shorter (*P* < 0.01), the median distance traveled by rats in the EP+ TAK-242 group was shorter (*P* < 0.01), the median distance traveled by rats in the EP+ TAK-242 group was shorter (*P* < 0.001), the total distance traveled by rats in the EP+ TAK-242 group was shorter (*P* < 0.001), and the median speed rats in of the EP+ TAK-242 group was slower (*P* < 0.001). Rats in EP+BAY 11–7082 and EP+TAK-242 improved by varying degrees (*n* = 10, ^*^P < 0.05; ^**^P < 0.01; ^***^P < 0.001).

## Discussion

TLE is one of the most common focal epilepsies in adolescents and adults (34). Approximately one-third of patients experience drug resistance; in patients with drug-resistant epilepsy and frequent seizures, there are varying degrees of learning, memory and mood disorders (35), which may be directly affected by the process of epilepsy (36), resulting in cognitive and emotional behavior problems in children (37). Treatment options for cognitive and mood disorders after epilepsy onset are limited, given that the mechanisms of TLE are unclear. Moreover, there are new requirements for pediatricians regarding treatment strategies for comorbidities that develop after epilepsy onset (38).

Microglias are an important bridge linking immunity and epilepsy. The role of microglias in epilepsy is gradually being recognized, and the TLR4 signaling pathway is considered to be one of the important bridges connecting innate immunity and acquired immunity (39). TLR4 is mainly expressed in microglias and mediates microglial activation (40). TLR4 activates the TIR domain containing adaptor IFN-β (TrIF)-related adaptor molecule (TRAM). The TRAM combines with TRIF to induce late activation of MAPK and NF-κB (41) and ultimately to increase cytokine expression and inflammatory damage (42, 43). NF-κB is a nuclear transcription factor closely related to immune regulation. However, the TLR4/NF-κB inflammatory signaling pathway has not been widely studied in epilepsy, and the role of neuroinflammation in epilepsy has gradually received attention. The control of the inflammatory response has been considered a conventional clinical treatment method and is expected to become a complementary therapy strategy in the future (44).

This study found that the expression of activated IBA-1 increased in microglial cells in rats in the epileptic group, with the cell volume increasing and amoeba-like and expression of the M1-type cytokine CD68 increasing. Pertinently, CD68 is a transmembrane glycoprotein expressed by human monocytes and tissue macrophages with phagocytic activity (45), and the expression of CD68 was increased in rats in the epileptic group. The expression of IBA-1 and CD68 decreased after treatment with the NF-κB inhibitor Bay11–7082 and the TLR4 inhibitor TAK-242. The protein levels of TLR4 and NF-κB were significantly increased in rats in the epileptic group compared with those of rats in the normal saline group. These results suggest excessive activation of NF-κB in microglias and activation of inflammatory pathways in pilocarpine-induced epileptic models. This activation trend was inhibited after treatment with the NF-κB inhibitor Bay11–7082 and the TLR4 inhibitor TAK-242, resulting in decreased expression of IBA-1 and CD68 proteins and decreased TLR4 and NF-κB proteins compared with that of rats in the epileptic group, suggesting that downregulating the TLR4/NF-κB pathway inhibits the activation of the inflammatory signaling pathway of microglias and produces antiepileptic effects.

Studies have shown that emotions occur in specific regions of several parts of the cerebral cortex, such as the amygdala, ventral striatum, putamen, caudate nucleus, and ventral tegmental area (46). TLE is more common in limbic system involvement, suggesting that children with TLE may be more prone to mood disorders (8, 47–49). In this study, The results were found in the open-field experiment that the retention time in the central region of rats in the EP group was delayed compared with that in rats in the control group, the locomotor distance traveled in the central region was prolonged, the total distance was prolonged, and the average speed was increased. Rats in the EP+Bay11–7082 and EP+TAK-242 groups improved to varying degrees after treatment. Results for rats in the EP group were significantly different from the those of rats in the control group, EP+BAY group and EP+TAK group. These data show that the inhibition of the TLR4/NF-κB inflammatory pathway can improve the emotional and behavioral disorders of epileptic rats. Studies have shown that a large number of hippocampal neurons die after epileptic seizure (25, 50–52), which may be related to microglias promoting epileptic-induced neurogenesis (53, 54). In the TUNEL experiment, neuronal apoptosis was greater in rats in the EP group, and the expression in rats in the two treatment groups, the EP+BAY group and EP+TAK group, was better than that of rats in the EP group. It is suggested that neuronal apoptosis after the epileptic seizure could be reduced by downregulating the TLR4/NF-κB inflammatory pathway. This may be caused by reducing the direct neuronal damage caused by inflammatory factors and increasing the neuronal damage caused by reducing phagocytosis to inhibit the activation of microglias, reduce excessive neurogenesis in the brain and alleviate brain injury after epileptic seizures.

## Conclusion

At the tissue level, the downregulation of the TLR4/NF-κB inflammatory pathway in epilepsy inhibited microglial activation and CD68 expression, inhibited hyperphagocytosis, inhibited the occurrence and exacerbation of epilepsy, and thus improved cognitive and emotional behavior after epilepsy. In addition, downregulation of the TLR4/NF-κB inflammatory pathway was proven by means of open-field experiments to improve cognitive and emotional behavior function after epileptic seizures in young rats.

## Data Availability Statement

The original contributions presented in the study are included in the article/supplementary material, further inquiries can be directed to the corresponding author.

## Ethics Statement

The animal study was reviewed and approved by the China Medical University Institutional Animal Care and Use Committee (No. 2017PS356K) approved all procedures.

## Author Contributions

QW, HW, and XL were involved in the conception and design of the study and the drafting of the manuscript. QW and YZ were involved in the analysis and interpretation of the data. YZ, XL, and JZ assisted in setting up the protocols as well as in their administration. QW performed all the experiments and acquired all data. All authors read and approved the manuscript.

## Funding

This study was supported by China Association Against Epilepsy-UCB Research Fund (No. 2019018).

## Conflict of Interest

The authors declare that the research was conducted in the absence of any commercial or financial relationships that could be construed as a potential conflict of interest.

## Publisher's Note

All claims expressed in this article are solely those of the authors and do not necessarily represent those of their affiliated organizations, or those of the publisher, the editors and the reviewers. Any product that may be evaluated in this article, or claim that may be made by its manufacturer, is not guaranteed or endorsed by the publisher.
